# A role of periaqueductal grey NR2B-containing NMDA receptor in mediating persistent inflammatory pain

**DOI:** 10.1186/1744-8069-5-71

**Published:** 2009-12-12

**Authors:** Jing Hu, Zhe Wang, Yan-Yan Guo, Xiao-Nan Zhang, Zhao-Hui Xu, Shui-Bing Liu, Hong-Ju Guo, Qi Yang, Fu-Xing Zhang, Xiao-Li Sun, Ming-Gao Zhao

**Affiliations:** 1 School of Pharmacy, Fourth Military Medical University, Xi'an 710032, China; 2 Department of Orthopaedics & Traumatology, Xijing Hospital, Fourth Military Medical University, Xi'an 710032, China; 3 Department of Anatomy, School of Basic Medical Science, Fourth Military Medical University, Xi'an 710032, China

## Abstract

The midbrain periaqueductal grey (PAG) is a structure known for its roles in pain transmission and modulation. Noxious stimuli potentiate the glutamate synaptic transmission and enhance glutamate NMDA receptor expression in the PAG. However, little is known about roles of NMDA receptor subunits in the PAG in processing the persistent inflammatory pain. The present study was undertaken to investigate NR2A- and NR2B-containing NMDA receptors in the PAG and their modulation to the peripheral painful inflammation. Noxious stimuli induced by hind-paw injection of complete Freund's adjuvant (CFA) caused up-regulation of NR2B-containing NMDA receptors in the PAG, while NR2A-containing NMDA receptors were not altered. Whole-cell patch-clamp recordings revealed that NMDA receptor mediated mEPSCs were increased significantly in the PAG synapse during the chronic phases of inflammatory pain in mice. PAG local infusion of Ro 25-6981, an NR2B antagonist, notably prolonged the paw withdrawal latency to thermal radian heat stimuli bilaterally in rats. Hyperoside (Hyp), one of the flavonoids compound isolated from *Rhododendron ponticum *L., significantly reversed up-regulation of NR2B-containing NMDA receptors in the PAG and exhibited analgesic activities against persistent inflammatory stimuli in mice. Our findings provide strong evidence that up-regulation of NR2B-containing NMDA receptors in the PAG involves in the modulation to the peripheral persistent inflammatory pain.

## Background

Brainstem descending pathways linking the periaqueductal gray (PAG), the rostral ventromedial medulla (RVM), and the spinal cord constitute a major mechanism in the modulation of pain transmission [1,2]. Considerable evidence has recently emerged regarding participation of this system in persistent pain conditions such as inflammation and neuropathy. Recent studies indicate that persistent pain after tissue or nerve injury is linked to an enhanced activation of descending modulatory circuits [2]. The increased excitability in the descending circuitry after injury likely reflects long-lasting changes in synaptic efficacy, similar to that seen in hippocampal synapses that are involved in learning and memory[3,4]. The increased net descending facilitatory drive leads to an amplification of the pain [2,5-8]. However, the cellular mechanisms underlying injury-induced synaptic plasticity in PAG circuitry are poorly understood.

Although PAG has limited direct projections to the spinal cord, it has a key role in the descending modulation of nociception and uses the RVM as an important intermediate in pain modulation, a site that projects directly to the spinal cord dorsal horn [5,9-11]. Glutamate and GABA play a critical role in processing pain at the PAG-RVM level [12].

Studies have shown that N-methyl-D-aspartate receptor (NMDAR) contribute to persistent pain following nerve and tissue injury [1,13]. Native NMDARs are composed of NR1, NR2 (A, B, C, and D), and NR3 (A and B) subunits. The formation of functional NMDARs requires a combination of NR1, an essential channel-forming subunit, and at least one NR2 subunit. NMDARs are highly expressed in the brain and subunit compositions may occur during early development and in different brain areas [14-16]. Previous studies show that peripheral inflammation increases the expression of NR2B-containing NMDARs and enhanced neurotransmitter release in the anterior cingulate cortex [4,17-19]. Inhibition of NR2B receptors by administering selective NR2B receptor antagonists locally into the anterior cingulate cortex or systemically, inhibits inflammation- related allodynia [17]. Subcutaneous formalin injection has been shown to produce an immediate increase of glutamate and aspartate in the spinal cord, and antagonists administered peripherally or spinally reduce pain following formalin injection [1,20]. NMDARs in PAG play a key role in the ascending transmission of pain [1,2,13,20,21]. However, little is known the role of NR2B-containing NMDARs in the PAG in processing prolonged peripheral inflammatory injury.

Hyperoside (Hyp), a flavonoid compound isolated from a folk remedy, *Rhododendron ponticum *L., which shows anti-inflammatory and analgesic activities [22]. However, little is known the mechanisms of Hyp underlying the persistent inflammatory pain processing. It is hopeful this study could shed light on the clinical treatment of chronic pain with traditional herbs.

In present study, we assessed the role of PAG NR2B receptor in processing of persistent inflammatory pain induced by left hindpaw injection of complete Freund's adjuvant (CFA). Our results demonstrate that NR2B receptor is up-regulated in the PAG and involves in the modulation to the prolonged peripheral injury.

## Results

### Up-regulation of NR2B receptors in the PAG after peripheral injury

**Figure 1:**
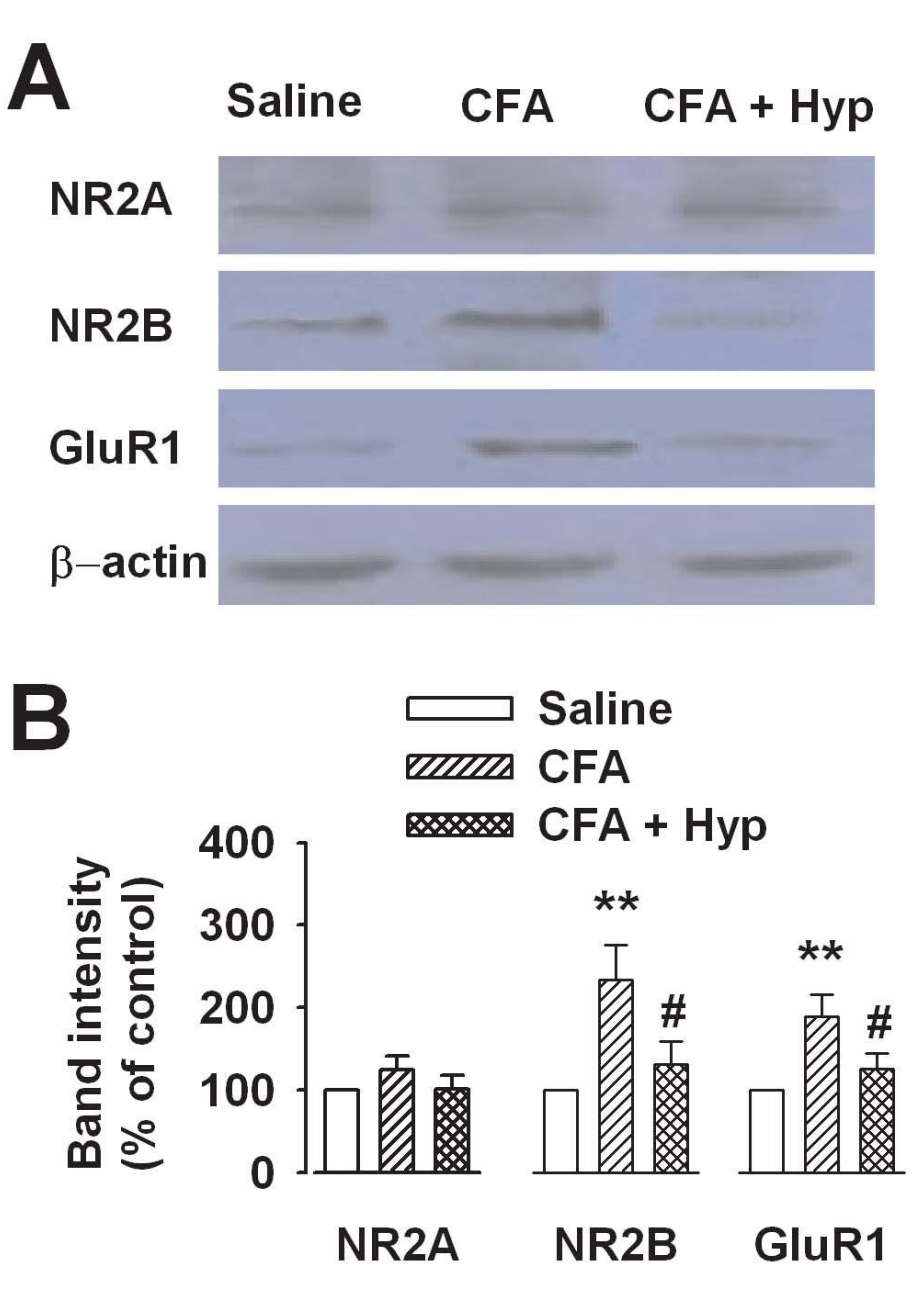
**Up-regulation of NR2B receptors in PAG after injury**. **(A) **Western blot results showed that the expression level of GluR1 and NR2B receptors, but not NR2A receptor, was increased in the PAG after CFA injection. Hyp reversed expression level of GluR1 and NR2B receptors. **(B) **Summary of expression of NR2A, NR2B, GluR1 receptors in the PAG after CFA-injection. Values were significantly different from saline control ** *p *< 0.01; from CFA-injected alone ^# ^*p *< 0.05.

Transgenic over-expression of NR2B-containing NMDARs in forebrain areas enhances inflammatory pain in mice [23]. Similarly, our previous results show that NR2B expression is increased in the anterior cingulated cortex after hindpaw CFA injection induced inflammatory pain [17]. As PAG plays important roles in processing pain-related information [1,6,20], it raises the possibility that NR2B expression is changed in the PAG after injury. To test this hypothesis, NR2B expression was examined by Western blot analysis on total PAG homogenates from mice. Western blot results showed that NR2B expression in PAG was notably increased at 3d after CFA-injection (234.4% ± 41.5% of saline, n = 6, *p *< 0.01; Fig. 1). Similar to NR2B, AMPA receptor GluR1 subunit expression was also increased after injury (189.4% ± 26.5% of saline, n = 6, *p *< 0.01; Fig. 1). However, no significant alteration was detected in the NR2A expression in the PAG after injury (Fig. 1). To further evaluate roles of NR2B receptor in inflammatory pain modulation, we treated CFA-injected mice with Hyp which shows anti-inflammatory and analgesic activities [22]. NR2B expression was significantly reduced in the PAG of CFA-injected mice administrated with Hyp (100 mg/kg, i.g. twice daily for 3 days) (132.1% ± 26.8% saline, n = 6, *p *< 0.05 *vs *CFA-injected alone; Fig. 1B). These findings imply that changes in NMDARs are selective for NR2B subunit in the PAG.

**Figure 2:**
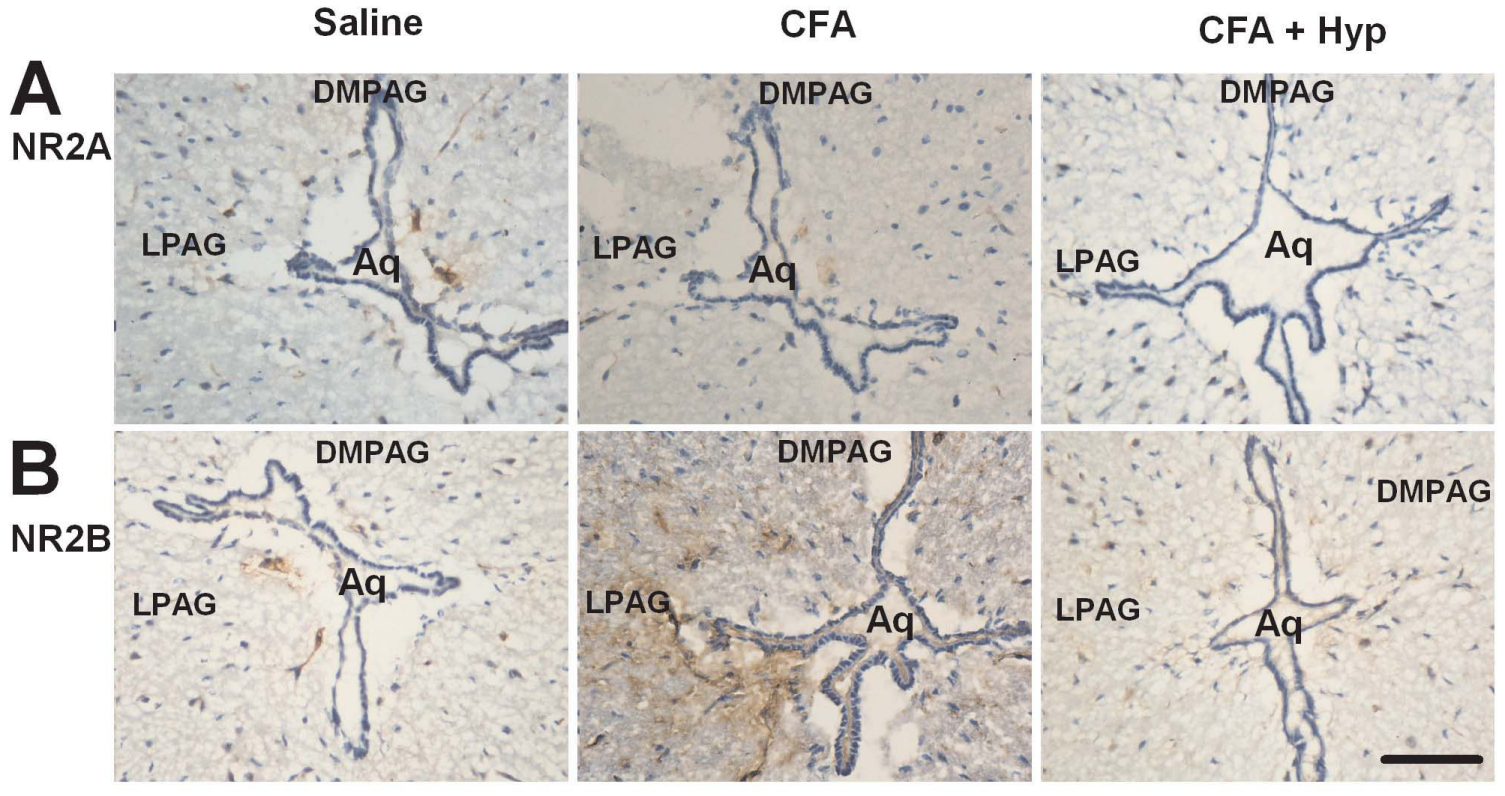
**Immunostaining of NMDA receptors in PAG**. Immunostaining was performed in the coronal midbrain slices containing the PAG. **(A) **Expression of NR2A in saline control, CFA, and CFA + Hyp treated mice. **(B)** Expression of NR2B in saline control, CFA, and CFA + Hyp treated mice. Scalebar (in B): A, B, 500 μM. DMPAG: Dorsomed periaqueductal gray; LPAG: Lateral periaqueductal gray.

To further confirm the selective changes of NMDA receptor subunits in PAG after CFA-injection, we performed immunostaining in the midbrain sections containing the PAG in mice. As shown in Fig. 2, expression of NR2B subunit was significantly increased in the LPAG of CFA-injected mice (n = 5) as compared with control mice (n = 6). Contrary to the NR2B, NR2A subunit was not significantly changed (n = 5). Administration of Hyp (100 mg/kg, i.g. twice daily for 3 days) reversed up-regulation of NR2B subunit in the LPAG of CFA-injected mice.

### Enhanced NMDAR-mediated synaptic transmission after injury

**Figure 3:**
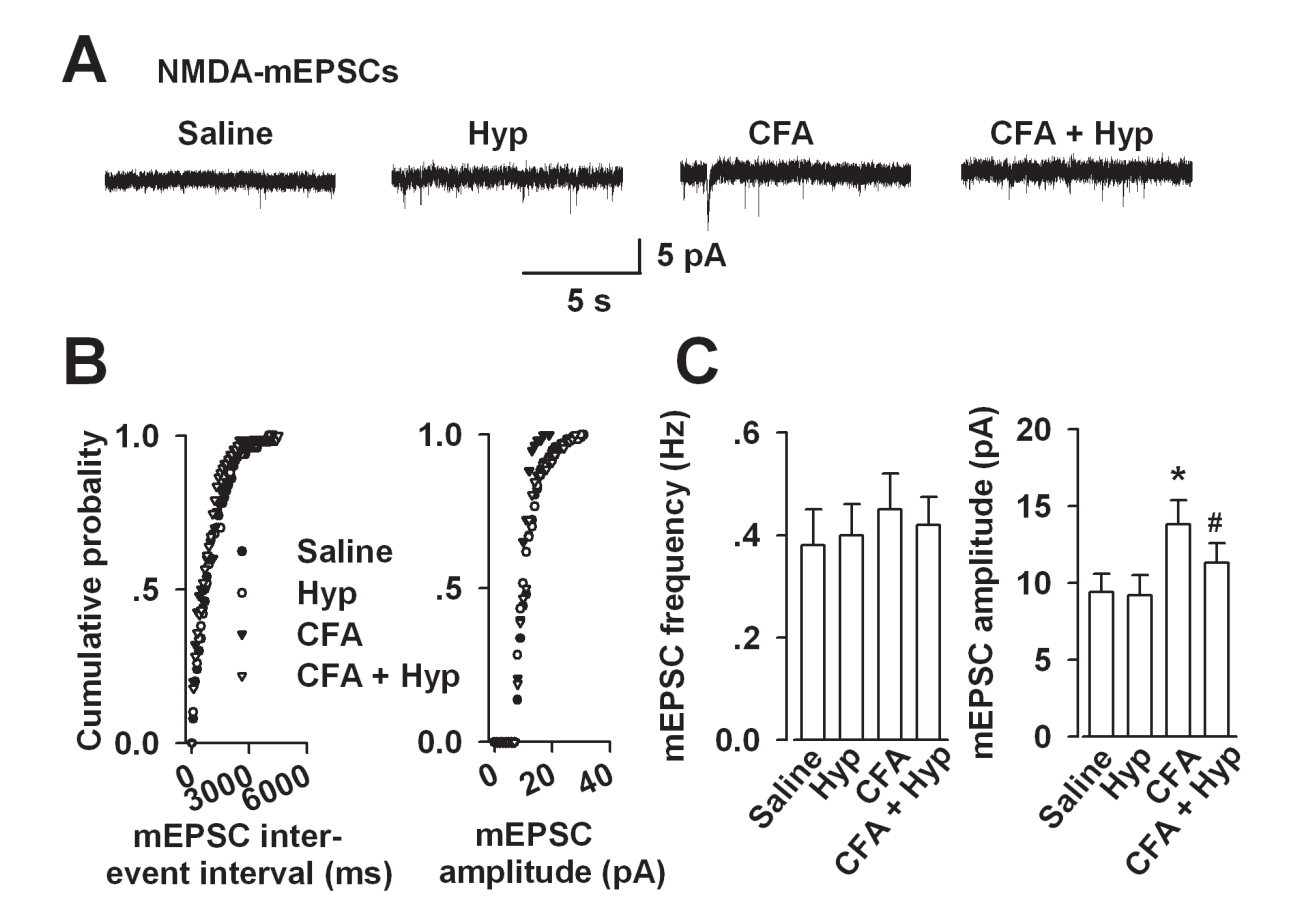
**Enhanced NMDA receptor-mediated transmission after injury**. **(A) **NMDA receptor-mediated mEPSCs recorded in PAG neurons at a holding potential of -30 mV. Representative traces show NMDA receptor-mediated mEPSCs in the saline, Hyp, CFA, and CFA + Hyp treated mouse. **(B) **Cumulative frequency (left) and amplitude (right) histogram of the mEPSCs from the cells in (A). Filled circles, from a saline control mouse; open circles, Hyp treated mouse; filled trangles, from a CFA-injected mouse; open trangles, from a CFA + Hyp treated mouse. **(C) **Summary of mEPSCs frequency (left) and amplitude (right) in neurons from the saline, Hyp, CFA, and CFA + Hyp treated mice. Values were significantly different from saline control * *p *< 0.05; from CFA-injected alone ^# ^*p *< 0.05.

To test whether up-regulation of NR2B receptor leads to changes in NMDAR-mediated synaptic transmission, we measured NMDAR-mediated miniature excitatory postsynaptic currents (mEPSCs) in the presence of CNQX (an AMPA receptor antagonist) in the lateral periaqueductal gray (LPAG). The amplitude, not the frequency of NMDAR-mediated mEPSCs was significantly increased at LPAG synapses of CFA-injected mice compared with saline controls (Frequency: saline, 0.38 ± 0.07 Hz, CFA, 0.45 ± 0.07 Hz, n = 8; amplitude: saline, 9.4 ± 1.0 pA, CFA, 13.8 ± 1.4 pA, n = 8, *p *< 0.05 *vs *saline; Fig. 3). As we predicted, Hyp reversed the enhanced NMDAR-mediated transmission in the PAG of CFA-injected mice (Frequency: 0.42 ± 0.05 Hz, n = 6; amplitude: 11.3 ± 1.1 pA, n = 8, *p *< 0.05 *vs *CFA-injected alone; Fig. 3). It suggests a possible mechanism for an enhancement in NMDAR function is an up-regulation of NR2B-containing NMDARs in the PAG.

### No changing of basal glutamatergic synaptic transmissionafter injury

**Figure 4:**
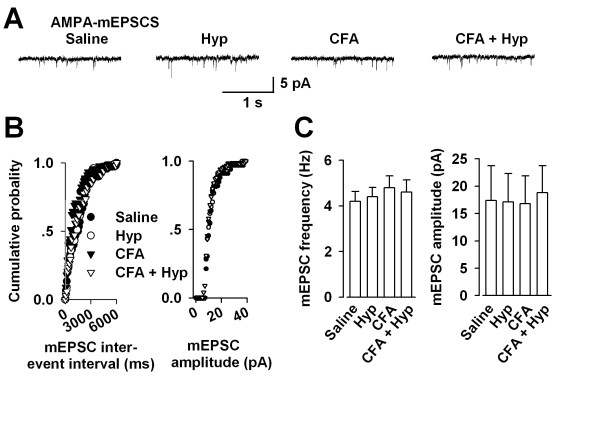
**Basal glutamatergic synaptic transmission in the PAG**. **(A) **AMPA receptor-mediated mEPSCs recorded in PAG neurons at a holding potential of -70 mV.Representative traces show AMPA receptor-mediated mEPSCs in the saline, Hyp, CFA, and CFA + Hyp treated mouse. **(B) **Cumulative frequency (left) and amplitude (right) histogram of the mEPSCs from the cells in (A). Filled circles, from a saline control mouse; open circles, Hyp treated mouse; filled trangles, from a CFA-injected mouse; open trangles, from a CFA + Hyp treated mouse. **(C) **Summary of mEPSCs frequency (left) and amplitude (right) in neurons from the saline, Hyp, CFA, and CFA + Hyp treated mice.

Previous studies show that hindpaw injection of CFA causes an enhancement in synaptic transmission in the ACC [19,24]. To determine whether nocuous stimuli affect basal glutamatergic synaptic transmission in the PAG, we measured the AMPA receptor-mediated mEPSCs. As shown in Figure 4, no significant alteration was detected in the AMPA receptor-mediated mEPSCs frequency and amplitude among the control, CFA-injected and Hyp treated mice (n = 6, Fig. 4). Results indicate that inflammatory painful stimuli have no effect on the basal excitatory synaptic transmission in the PAG.

### Behavioral responses to inhibition of NMDARs

**Figure 5:**
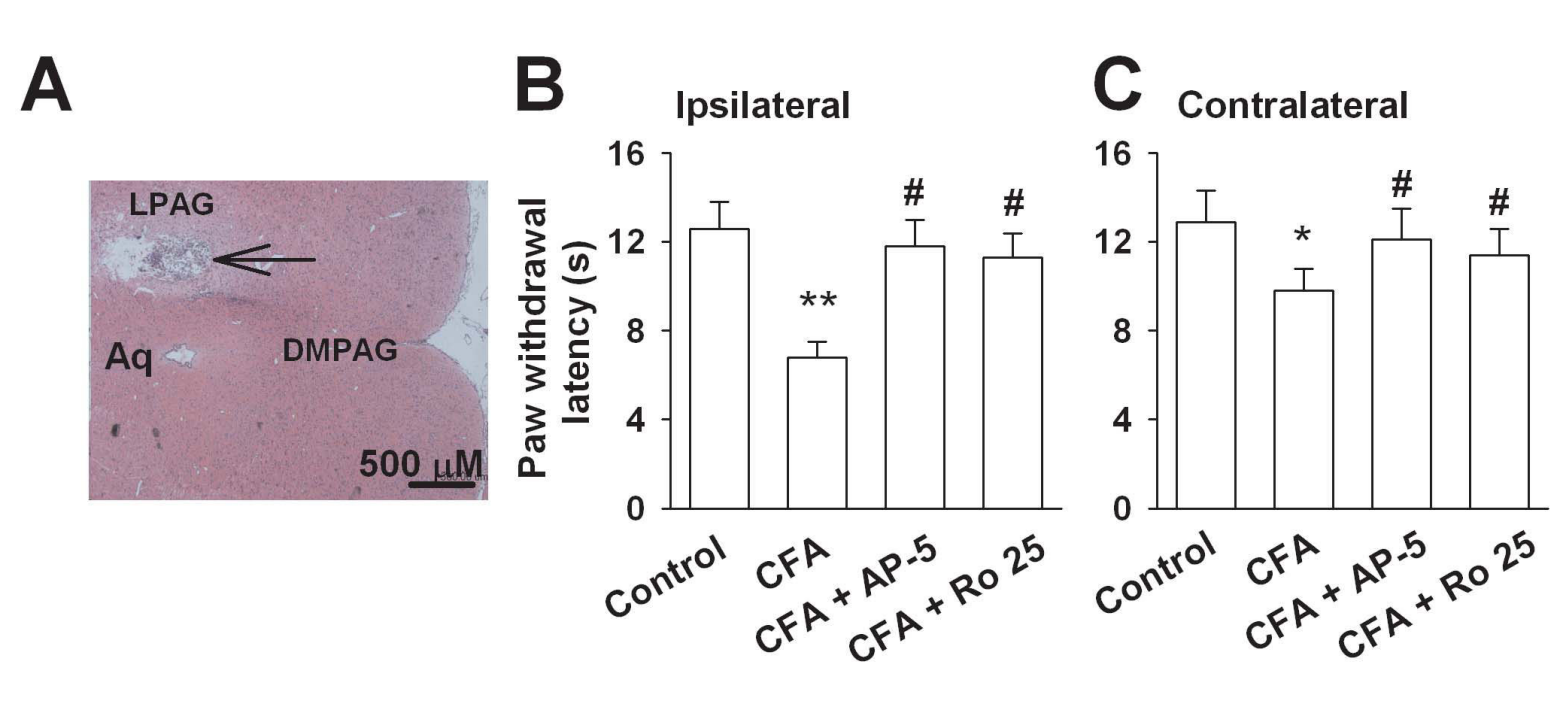
**Changes in the PWL by AP-5 and Ro 25-6981**. **(A) **Representative coronal section of rat midbrain showing PAG injection sites. Scale bar, 500 μm. Aq: aqueduct; DMPAG: Dorsomed periaqueductal gray; LPAG: Lateral periaqueductal gray. **(B) **PWL in the CFA-treated paws (ipsilateral) by AP-5 and Ro25-6981 infusion. **(C) **PWL in the right hind paws (contralateral) by AP-5 and Ro25-6981 infusion. Values were significantly different from saline control * *p *< 0.05, ** *p *< 0.01; from CFA-injected alone ^# ^*p *< 0.05.

Our results show that NR2B expression is increased in the PAG after peripheral injury, suggesting that inhibition of the NR2B subunit may alter inflammation -induced behavioral responses. To test this hypothesis, we microinjected AP-5 (an NMDAR antagonist) and Ro 25-6981 (a selective NR2B antagonist) into the PAG of rats and evaluated the effects on thermal hyperalgesia induced by CFA-injection (Fig. 5A). The paw withdrawal latency (PWL) to thermal radian heat stimuli is a widely used nociceptive measure to study hyperalgesic behavior. As illustrated in Fig. 5, CFA-treated ipsilateral paws (left) showed a significant decrease in PWL when compared to left paws of control rats (from 12.6 ± 1.2 s to 6.8 ± 0.7 s, n = 10, *p *< 0.01 *vs *CFA-injected alone; Fig. 5B). Interestingly, PWL in contralateral paws (right) also showed a slight decrease when compared to right paws of control rats (from 12.9 ± 1.4 s to 9.8 ± 1.0 s; n = 11, *p *< 0.05 *vs *CFA-injected alone; Fig. 5C). Microinjection of Ap-5 (0.5 μg/0.5 μl) into the PAG induced a significant increase in PWL in CFA injected rats (ipsilateral: 11.8 ± 1.2 s, n = 5, *p *< 0.05 *vs *saline microinjection, Fig. 5B; contralateral: 12.1 ± 1.4 s, n = 5, *p *< 0.05 *vs *saline microinjection, Fig. 5C). Similar results were observed for PWL with the microinjection of Ro 25-6981(0.5 μg/0.5 μl) into the PAG (ipsilateral: 11.2 ± 1.1 s, n = 5, *p *< 0.05 *vs *saline microinjection, Fig. 5B; contralateral: 11.5 ± 1.6 s, n = 5, *p *< 0.05 *vs *saline microinjection, Fig. 5C).

### Inhibiting thermal hyperalgesia by Hyp

**Figure 6:**
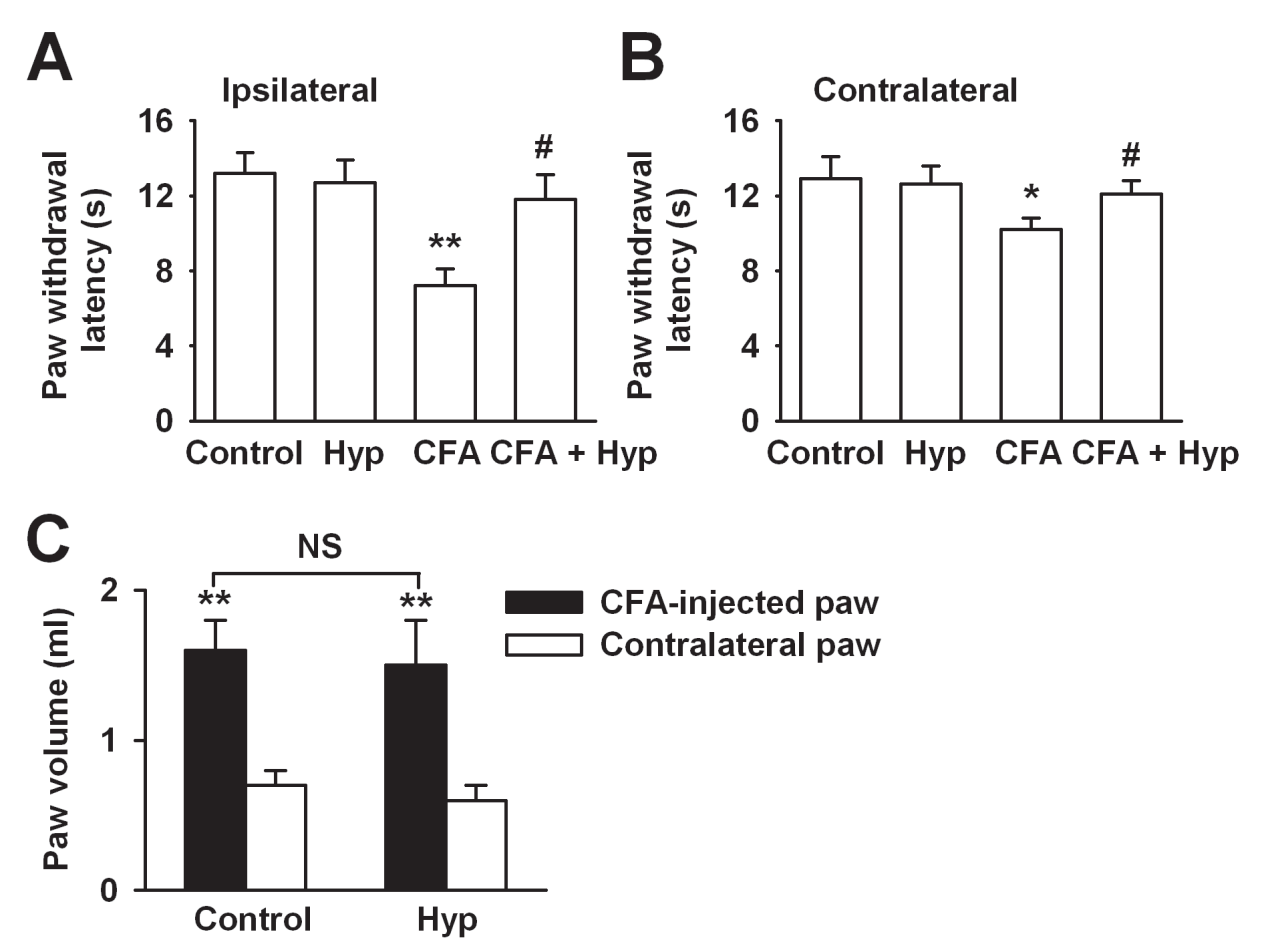
**Changes in the PWL by Hyperoside**. **(A) **PWL in the CFA-treated paws (ipsilateral) from the control, Hyp, CFA + saline, and CFA + Hyp treated mice. **(B) **PWL in the right hind paws (contralateral) from the control, Hyp, CFA + saline, and CFA + Hyp treated mice. Values (in A and B) were significantly different from saline control * *p *< 0.05, ** *p *< 0.01; from CFA-injected alone, # p < 0.05. **(C) **Paw volume in the CFA-treated and contralateral paws from control and Hyp treated mice. Values (in C) were significantly different from contralateral paws ** *p *< 0.01.

As illustrated in Fig. 1 and Fig. 2, administration of Hyp reversed up-regulation of NR2B receptors in the PAG of CFA-injected rats, it is reasonable to predict Hyp inhibiting the thermal hyperalgesia induced by chronic inflammation. As shown in Fig. 6, administration of Hyp (100 mg/kg, i.g. twice daily for 3 days) alone did not change PWL in hindpaw saline-injected rats. However, Hyp could significantly reverse ipsilateral PWL (from 7.2 ± 0.9 s to 11.8 ± 1.3 s; n = 8, *p *< 0.05 *vs *CFA-injected alone; Fig. 6B) and contralateral PWL (from 10.2 ± 0.6 to 12.1 ± 0.7 s; n = 8, *p *< 0.05 *vs *CFA-injected alone; Fig. 6B) in CFA-injected rats.

To test whether analgesic effect of Hyp is associated to its peripheral anti-inflammatory actions, we evaluate degree of edema by measuring the volume of hindpaw. Paired t-test showed a significant increase in the volume of the CFA-injected paws when compared to contralateral paws (inflamed paws: 1.6 ± 0.2 ml, contralateral paws: 0.7 ± 0.1 ml, n = 8, *p *< 0.01; Fig. 6C). However, administration of Hyp (100 mg/kg, i.g. twice daily for 3 days) had no effects on the volume of the CFA-injected paws (CFA: 1.6 ± 0.2 ml, CFA + Hyp:1.5 ± 0.3 ml, n = 8; Fig. 6C).

## Discussion

PAG and adjacent structures constitute a pain-control system that descends from the brain onto the spinal cord. Considerable evidence has recently emerged regarding participation of this system in persistent pain conditions such as inflammation and neuropathy [2,5-8,25-27]. An increasing number of studies have shown that the descending system can facilitate spinal transmission of pain signals during persistent nociception. Anatomical, electrophysiological and pharmacological evidence has established the RVM as an integral relay in descending modulation of nociception, including that elicited by PAG stimulation [5,10,28]. Fields et al. [29] have characterized ON and OFF cells in the RVM that may constitute the physiological basis for generation of bidirectional modulation of spinal nociceptive transmission. Off-cells are proposed to contribute to inhibitory influences, while ON-cells are proposed to contribute to facilitatory influences that descend from the RVM. Projections from PAG modulate the state of ON or OFF cells in RVM and trigger descending facilitation or inhibition [6].

NMDARs in the cortex, PAG, and RVM play a key role in the ascending transmission of pain [1,2,12,13,20,21]. Recently, chemical or electrical stimulation in the rat anterior cingulate cortex (ACC), which sends projections to the PAG and thus communicates indirectly with the RVM, enhanced the tail-flick reflex [18,30-32]. Persistent peripheral inflammation leads to enhanced presynaptic glutamate release in the ACC [19,33,34]. Microinjection of NR2B antagonist into the ACC significantly reduces mechanical allodynia induced by hindpaw CFA-injection [17]. Microinjection of NMDA into the RVM facilitate the tail-flick reflex in a dose-dependent manner, an effect blocked by the NMDAR antagonist AP-5 [35,36]. However, little is known the roles of NR2B-containing NMDARs in the PAG in processing the peripheral persistent inflammatory pain.

In present investigation, we demonstrate for the first time that changes in NMDARs expression in the PAG is selective for NR2B subunits, not NR2A ones. The results demonstrate a significant increase in NR2B expression in the lateral areas of the PAG (LPAG). Up-regulation of NR2B is verified by the recordings of NMDAR-mediated mEPSCs. It is reasonable to conclude that the enhancement in NMDARs is due to the up-regulation of NR2B-containing NMDARs in the PAG. Different reports have shown that phosphorylation of the NMDAR NR1 subunit is associated with noxious stimulation demonstrated by capsaicin, carrageenan and formalin injection to rodents [37-39]. We do not exclude the possibility that NMDAR NR1 subunit is altered in our experiment condition.

The increased descending facilitatory drive from the PAG and RVM leads to an amplification of the pain. Hindpaw CFA-injection induced inflammation is an animal model for persistent inflammatory pain which nociceptive pathway involves anatomical sites at the level of the brain, spinal cord, and periphery. As shown in many other studies the inflamed ipsilateral paws show a significant decrease in PWL than control paws [40,41]. Also, the contralateral paws of CFA-injected group show a slight decrease in PWL than control paws, even though the decrease in PWL of contralateral paws is much smaller than that on the ipsilateral side [42]. This indicates that in chronic inflammatory pain condition the decrease in PWL to thermal stimuli is not confined only to the CFA-inoculated paws but also affects the non-inoculated contralateral paws. Consistent with this report, we find that unilaterial CFA injection causes a bilateral decrease in PWL in present study. PAG infusion of NR2B antagonist, Ro 25-6981, leads to prolong PWL bilaterially, suggesting the NR2B-containg NMDARs in the PAG modulate the thermal hyperalgesia to peripheral inflammatory injury.

However, it is important to define in which circumstances the descending system is inhibitory and in which it is facilitatory. The problem is that, even for similar types of experiment, some laboratories conclude that the descending system has an inhibitory role in nociception while other laboratories conclude that it has a facilitatory role [5,6,10,28]. The similar contradiction is observed in the experiments to define the role of NMDARs in the PAG in descending system. In some experiments show that activation of NMDARs in the PAG causes an inhibition of pain response [21,43,44], while others show that NMDARs may play a critical role in induction of spinal hyperalgesia after prolonged noxious stimulation [1,2,20]. One way to solve this apparent contradiction is to hypothesize that experiments are performed in different conditions, such as intense of noxious stimuli (acute or chronic), types of injury (inflammatory or neurophathic), time points after induction of injury.

Interestingly, present results indicate that basal excitatory synaptic transmission is not altered in the LPAG synapse following exposure to peripheral inflammation. This is demonstrated by unchanged AMPA receptor-mediated mEPSCs frequency and amplitude in PAG slice recordings. However, GluR1 subunits were found over-expressed in the PAG by Western-Blot detection. At least two possible mechanisms may explain this difference. First, mEPSC recordings could only detect the surface expression of AMPA receptors while Western-blot analysis indicates the total APMA receptors including the cytoplastic distribution. Previous reports suggest that synaptic delivery of the GluR1 subunit from extrasynaptic sites and cytoplasm is the key mechanism underlying synaptic plasticity and pain recessing [33,45-47]. Second, mEPSC recordings were performed in the LPAG while the tissues for Western-blot analysis were from the whole PAG in present study.

To further confirm the selective changes of NMDARs subunits in PAG after CFA-injection, we treated the CFA-injected mice with Hyperoside (Hyp), a flavonoid compound isolated from a folk remedy, which shows anti-inflammatory and analgesic activities [22]. In the present study, we found Hyp significantly reversed the up-regulated NR2B receptors in the PAG from mice with peripheral injury, whereas it did not affect the NR2B receptor expression in the normal physiological conditions. The down-regulated NR2B receptor is quite correlated with its analgesic effects on the thermal hyperalgesia induced by chronic inflammation. When evaluated its peripheral anti-inflammatory activities, we found that Hyp had marginal effect on the paw edema by CFA-injection. The data suggest analgesic effects of Hyp involving the modulation of synaptic transmission at the level of PAG. However, we could not exclude the possible effects of Hyp on the pain transmission through modulation of algesic mediators, e.g. TNF, substance P, bradykinin, or serotonin.

Taken together, our findings provide strong evidence that up-regulation of NR2B-containing NMDARs in the PAG involves in the modulation to the peripheral injury. It is hopeful this study could help further understand NR2B-containing NMDARs function during chronic pain processing and descending facilitation.

## Methods

### Animals

Six- to eight-week-old male C57BL/6 mice and Sprague Dawley rats were used in the experiments. All animal protocols used were approved by the Animal Care and Use Committee of the Fourth Military Medical University. Animals were housed under a 12 h light/dark cycle with food and water provided *ad libitum*. To induce inflammatory pain, 10 μl (for mouse) or 50 μl (for rat) of 50% CFA (Sigma, St. Louis, MO) was injected subcutaneously into the dorsal surface of left hindpaw. The degree of edema was evaluated by measuring hindpaw volume using a plethysmometer (UgoBasile, Varese, Italy). All experiments were performed 3-5 days after hindpaw CFA-injection.

### Western blot

Equal amounts of protein (50 μg) from the PAG of mice were separated and electrotransferred onto PDVF membranes (Invitrogen), which were probed with anti-NR2A, anti-NR2B, anti-GluR1 (Chemicon), and with β-actin (Sigma) as a loading control. The membranes were incubated with horseradish peroxidase conjugated secondary antibodies (anti-rabbit IgG for the primary antibodies), and bands were visualized using an ECL system (Perkin Elmer).

### Immunohistochemistry

Immunostaining was performed as described previously [48]. Briefly, the PAG sections from mice were first treated with 0.75% Triton X-100 and 1% H_2_O_2 _in PBS for 1 h, and then processed for 30 min in 3% normal goat serum, followed by incubation with anti-NR2A and anti-NR2B, anti-GluR1 (Chemicon) monoclonal antibody overnight at room temperature. Secondary reactions with biotinylated goat anti-mouse immunoglobulin (Vector Laboratories) for 1 h were followed by avidin- biotin-peroxidase complexes (Vector Laboratories) for 1 h. A nickel-intensified diaminobenzidine with glucose oxidase was used as the final chromogen. Sections were washed several times, mounted on gelatinized slides, dehydrated through a series of ethanol solutions, cleared in xylene, and covered with glass coverslips. Controls, performed by replacing primary antibody with 1% NGS in the protocol.

### Slice preparation and Whole-cell patch-clamp recordings

Coronal brain slices (300 μm), containing the PAG, were prepared as described [49,50]. Slices were transferred to submerged recovery chamber with oxygenated (95% O_2 _and 5% CO_2_) artificial cerebrospinal fluid (ACSF) containing (in mM): 124 NaCl, 2.5 KCl, 2 CaCl_2_, 2 MgSO_4_, 25 NaHCO_3_, 1 NaH_2_PO_4_, 10 glucose at room temperature for at least 1 h. Experiments were performed in a recording chamber on the stage of an Axioskop 2FS microscope with infrared DIC optics for visualization of whole-cell patch-clamp recording. Miniature excitatory postsynaptic currents (mEPSCs) were recorded from LPAG neurons with an Axon 200B amplifier (MDS, Inc., CA). AMPA receptor-mediated mEPSCs were recorded from the neurons clamped at -70 mV. The recording pipettes (3-5 MΩ) were filled with solution containing (mM) 145 K-gluconate, 5 NaCl, 1 MgCl_2_, 0.2 EGTA, 10 HEPES, 2 Mg-ATP, and 0.1 Na_3_-GTP (adjusted to pH 7.2 with KOH). The NMDAR-mediated component of mEPSCs was pharmacologically isolated in ACSF containing CNQX (20 μM), glycine (1 μM), TTX (0.5 μM). The patch electrodes contained 102 mM cesium gluconate, 5 mM TEAchloride, 3.7 mM NaCl, 11 mM BAPTA, 0.2 mM EGTA, 20 mM HEPES, 2 mM MgATP, 0.3 mM NaGTP, and 5 mM QX-314 chloride (adjusted to pH 7.2 with CsOH). Neurons were voltage clamped at -30 mV. Picrotoxin (100 μM) was added in the ACSF for the all recordings. Access resistance was 15-30 MΩ and monitored throughout the experiment. Data were discarded if access resistance changed more than 15% during an experiment.

### Surgery and microinjection

Cannula implantation was performed according to the procedure by Ghelardini [20]. Rats were implanted with 7-mm stainless steel guide cannula (23-gauge) into the right sige of PAG (6.8 mm posterior to Bregma, 0.5 mm lateral from the midline, 4.5 mm beneath the surface of the skull) with the guide cannula angled 26° to the vertical. The dummy cannulas, cut 0.5 mm longer than guide cannulas, were inserted into the guide cannulas to prevent clogging and reduce the risk of infection. Rats were given at least 5 days to recover before experimentation. A 30-gauge injection cannula that was 1.5 mm lower than the guide was used for drug infusion. AP-5 (0.5 μg/0.5 μl), Ro25-6981 (0.5 μg/0.5 μl) or saline was delivered at the rate of 0.5 μl/min using a pump. After infusion, the cannulas were left in place for an additional 2 min to allow the solution to diffuse away from the cannula tip.

### Measurement of thermal hyperalgesia

The method of Hargreaves et al. (1988) was used to assess paw withdrawal latency (PWL) to a thermal nociceptive stimulus [41]. To assess thermal nociceptive responses, a commercially available plantar analgesia instrument (BME410A, Institute of Biological Medicine, Academy of Medical Science, China) was used. Animals were placed in individual plastic boxes and allowed to adjust to the environment for 1 h. Thermal hyperalgesia was assessed by measuring the latency of paw withdrawal in response to a radiant heat source. Rats were housed individually into Plexiglas chambers on an elevated glass platform, under which a radiant heat source was applied to the plantar surface of the hind paw through the glass plate. The heat source was turned off when the rat lifted the foot, allowing the measurement of time from onset of radiant heat application to withdrawal of the rat's hindpaw. This time was defined as the PWL. Both paws were tested alternately at 5-min intervals for a total of five trials. A 20 s cutoff was used to prevent tissue damage. Both hindpaw was tested independently with 10 min interval between trials.

### Histological identification

To confirm the locations of the intra-PAG injection sites, brains were fixed with 4% paraformaldehyde and dehydrated through an ascending alcohol series. The rat brains coronal sections (30 μm) were mounted on glass slides and stained with hematoxylin and eosin. Images were taken using an Olympus light microscope equipped with a CCD camera.

### Data analysis

Results were expressed as mean ± SEM. Statistical comparisons were performed using a t-test, One-way ANOVA, or Two-way Repeated Measures ANOVA with student's t-test for post-hoc. In all cases, *p *< 0.05 was considered statistically significant.

## List of abbreviations

ACSF: artificial cerebrospinal fluid; AMPA: α-amino-3-hydroxy-5-methyl-4-isoxazolepropionic acid; Aq: aqueduct; CFA: complete Freunds adjuvant; DMPAG: Dorsomed periaqueductal gray; Hyp: Hyperoside; LPAG: Lateral periaqueductal gray; mEPSC: miniature excitatory postsynaptic current; NMDAR: N-methyl D-aspartate receptor; PAG: periaqueductal grey; RVM: rostral ventromedial medulla.

## Competing interests

The authors declare that they have no competing interests.

## Authors' contributions

JH, XNZ and QY are responsible for performance of Western-blot and immunostaining. ZW, HJG, SBL and FXZ are responsible for performance of behavioral test. YYG and ZHX are responsible for performance of electrophysiology. XLS and MGZ are responsible for experimental design and writing the manuscript. All authors read and approved the final manuscript.
